# Investigation of Polymer Gel Reinforced by Oxygen Scavengers and Nano-SiO_2_ for Flue Gas Flooding Reservoir

**DOI:** 10.3390/gels9040268

**Published:** 2023-03-24

**Authors:** Wenli Qiao, Guicai Zhang, Ping Jiang, Haihua Pei

**Affiliations:** School of Petroleum Engineering, China University of Petroleum (East China), Qingdao 266580, China

**Keywords:** flue gas, reinforced gel, oxygen scavenger, nano-SiO_2_, degradation, long-term stability

## Abstract

Polymer gel plugging is an effective technique for gas mobility control in flue gas flooding reservoirs. However, the performance of polymer gels is extremely susceptible to the injected flue gas. A reinforced chromium acetate/partially hydrolyzed polyacrylamide (HPAM) gel, using thiourea as the oxygen scavenger and nano-SiO_2_ as the stabilizer, was formulated. The related properties were evaluated systematically, including gelation time, gel strength, and long-term stability. The results indicated that the degradation of polymers was effectively suppressed by oxygen scavengers and nano-SiO_2_. The gel strength would be increased by 40% and the gel kept desirable stability after aging for 180 days at elevated flue gas pressures. Dynamic light scattering (DLS) analysis and Cryo-scanning electron microscopy (Cryo-SEM) revealed that nano-SiO_2_ was adsorbed on polymer chains by hydrogen bonding, which improved the homogeneity of gel structure and thus enhanced the gel strength. Besides, the compression resistance of gels was studied by creep and creep recovery tests. The failure stress of gel with the addition of thiourea and nanoparticles could reach up to 35 Pa. The gel retained a robust structure despite extensive deformation. Moreover, the flow experiment indicated that the plugging rate of reinforced gel still maintained up to 93% after flue gas flooding. It is concluded that the reinforced gel is applicable for flue gas flooding reservoirs.

## 1. Introduction

Flue gas, which contains N_2_ (80–85%), CO_2_ (15–20%), and a small amount of O_2_, is an eco-friendly and effective gas source in gas flooding reservoirs [1,2]. However, the injected flue gas tends to flow towards the large channel subjected to the heterogeneity of the reservoir, greatly reducing the sweep efficiency and thus decreasing the recovery [3,4]. Polymer gel treatment is a productive method for controlling the flow of N_2_ and CO_2_ [5,6,7]. Gel performance, including gelation performance and long-term stability, is often adopted to estimate the implementation effect of polymer gels for controlling gas flow [8,9,10]. Our previous studies have shown that flue gas prolongs the gelation time and reduces the long-term stability of organic chromium gels [11]. In detail, CO_2_ in flue gas can reduce the pH value of the polymer solution and prolong the gelation time. O_2_ leads to the degradation of the polymer, which in turn undermines the long-term stability of the gel. Thereafter, the performance of resorcinol/hexamethylenetetramine-based polymer gel in flue gas environments was studied [12]. The results show that flue gas promotes gelation performance while seriously threatening the long-term stability of gel. Therefore, it is of great value to improve the gel’s performance in a flue gas environment. Currently, the improvement methods for hash reservoir conditions have been studied by many researchers. However, there are few reports related to the flue gas flooding reservoir [10].

Previous studies show that the addition of a high-efficiency oxygen scavenger can significantly reduce the dissolved oxygen content, scavenge free radicals, and improve the stability of the polymer [13]. Generally, there are three kinds of oxygen scavengers based on the scavenging mechanism, including oxygen removal, radical scavengers, and sacrificial agents. Dithionite and sulfite are efficient oxygen removal agents to reduce the dissolved oxygen content. However, on account of the extremely active redox system and the potential source of oxygen, they can be harmful to the polymer stability [14].

The degradation mechanism of the polymer is a kind of free radical reaction. The polymer radical rapidly interacts with oxygen and forms the peroxy radical again. Radical scavengers are generally employed to inhabit the free radical reaction because they provide protons that can trap radical species and thus form new stable radicals. Efficient radical scavengers insist on phenylphosphonic acid, butylated hydroxytoluene, thiourea, sodium thiocyanate, mercaptobenzothiazole, mercaptobenzimidazole, and so on [15]. Owing to the unstable hydrogens in alcohol molecules, alcohols are usually adopted as sacrificial agents to prevent radical degradation of polymers [16]. However, sometimes the incorporation of alcohol may be counterproductive. Studies have reported that the addition of sacrificial agents to radical scavengers is an effective solution to restrain the viscosity loss of polyacrylamide. Gaillard et al. and Wellington studied the effects of thiourea and isopropanol as free radical scavengers and sacrificial agents on the anti-oxidative degradation of polymers. It is claimed that, at 90 °C and 100 °C, the combination of thiourea and isopropanol effectively inhibited the free radical degradation induced by dissolved oxygen, and the viscosity of the solution was maintained. However, when the temperature increased to 110 °C, the viscosity of the solution decreased significantly, indicating that thiourea and isopropanol could not effectively prevent the degradation of the polymer at higher temperatures (>110 °C) [15,17].

As a widely used free radical scavenger in oilfields, thiourea tries to clean up the free radicals and intercepts the free radical reaction chains, which reduce the risk of polymer degradation [18]. The reaction mechanism between thiourea and O_2_ is as follows:

O_2_ → O• (1)

O_2_ + thiourea → O• (2a)

O_2_ + O• + thiourea → O_2_ (excessive thiourea) (2b)

O_2_ + O•+ thiourea → O• (excessive O_2_) (2c)

O•+ P → P_1_ • (3)

The relative amounts of O_2_ and thiourea in the polymer solution determine the direction of the radical reaction. An intermediate transition band can be formed between O_2_ and thiourea, which reduces the activation energy of O_2_ cleavage to O• and makes it easier to produce O•. Therefore, thiourea makes the generation of O• faster. When in the presence of excessive thiourea in the system, it acts as an effective free radical scavenger, reacting according to Equation (2b) and inhibiting the generation of free radicals. When in the presence of excessive O_2_, it reacts according to Equation (2c) and promotes the generation of free radical O•, which may lead to free radical-induced polymer degradation [16].

At present, the performance of gels is commonly advanced by adding stabilizers, among which the addition of the nanoparticle is an efficient way [19,20,21,22,23,24]. In 2006, Portehault et al. [25] added nano-SiO_2_ with a particle size of 20 nm into n-isopropylacrylamide and found that the side chains of N-isopropylacrylamide could be adsorbed on the SiO_2_ surface, forming an elastic network structure. Zhu et al. [26] reported that the viscosity of hydrophobically associative polyacrylamide aqueous solution as well as the temperature and salt resistance of the polymer could be increased by the addition of SiO_2_ nanoparticles. It is claimed that the nanoparticles adsorb on the polymer chain and interact with the polymer groups, inhibiting the degradation of the polymer [22,27]. Moreover, nano-SiO_2_ greatly enhances gel stability and can be used in harsh conditions, for example, in high salinity and high temperature reservoirs [28]. Additionally, the improvement of gel performance is mainly determined by the types and concentration of nanoparticles [29,30,31]. Dai et al. [32] reported that silica nanoparticles (15 nm) effectively shortened gelation time and enhanced the gel strength under the salinity of 23 × 10^4^ mg/L and a temperature of 110 °C. Furthermore, the degree of syneresis is also a key criterion to evaluate the stability of gels. Serious syneresis behavior threatens the long-term stability and lowers gel treatment efficiency. This problem can be ameliorated with the addition of nanoparticles due to the enhancement in the water-holding capacity of gels [33,34]. Polymers can be bridged with nanoparticles by hydrogen bonding between the silyl alcohol group (Si–OH) in nano-SiO_2_ and the amide group (–CONH_2_) as well as hydroxyl group (–OH) in polymers, significantly enhancing the stability of gels [22,27,35].

In this paper, thiourea and nano-SiO_2_ were used as additives to improve the stability of polymer gels suitable for flue gas flooding reservoirs. Extensive experimental studies were carried out on the properties of gels by rheological testing, dynamic light scattering (DLS), Fourier transform infrared spectroscopy (FTIR), and Cryo-scanning electron microscopy (Cryo-SEM) with energy dispersive spectroscopy (EDS). In addition, the compression resistance of polymer gels was studied by creep test. The plugging capability was measured by flooding experiments and visual glass model experiments, and then an appropriate gel injection volume was designed for field application in the Xinjiang oilfield.

## 2. Results and Discussion

### 2.1. Oxygen Scavenger Reinforced Polymer Gel

#### 2.1.1. Stability of Polymer

**Figure 1:**
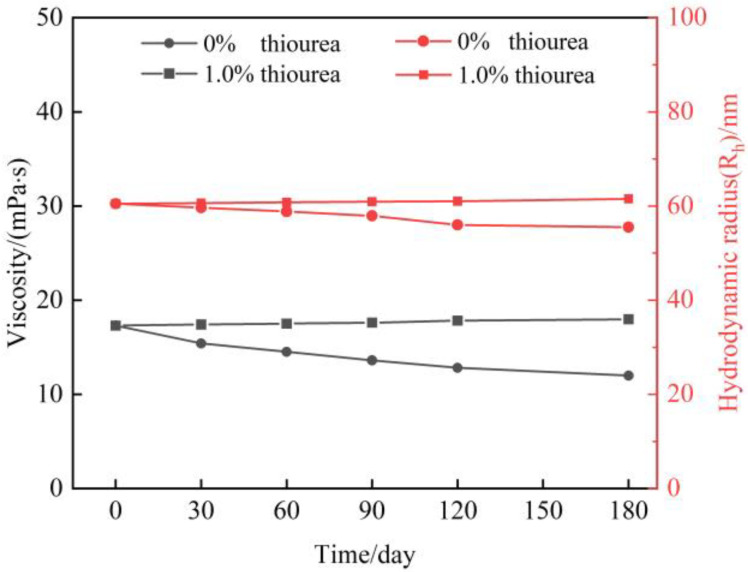
Properties of HPAM with the addition of thiourea in high pressure of flue gas.

In gas flooding reservoirs, it is generally required that the polymer gel maintain long-term stability and that the validity period for polymer gel treatment needed to reach up to 180 days. Meanwhile, high-pressure flue gas will be continuously injected into the reservoir during flue gas flooding. However, the flue gas contains 3% O_2_, and under high pressure flue gas, O_2_ will be continuously dissolved in large quantities in the gels. Our previous work shows that O_2_ leads to polymer degradation and impairment of the polymer stability [11,12]. Hence, a sufficient amount of thiourea needs to be added to the gelant to meet the long-term stability of the polymer. In this study, a 0.5 wt% HPAM solution is prepared. 1.0 wt% thiourea is optimized and added to the polymer solution. The effect of thiourea on polymer stability in a flue gas environment is studied. In particular, the pressure of flue gas was set at 6 MPa owing to that it is a relatively high injection pressure for flue gas flooding. It also should be emphasized that all the experiments are conducted at 42 °C (reservoir temperature). Figure 1 displays the result of viscosity and R_h_ of the polymer solution as a function of aging time. It is observed that the viscosity of polymers without the addition of thiourea decreases obviously within 180 days, which is the same tendency for hydrodynamic radius (R_h_). The decrease percentage of viscosity is calculated to be up to 30% after aging for 180 days, and 28% in the R_h_. By contrast, the polymer with the addition of 1 wt% thiourea shows slightly change in viscosity and R_h_ after 180 days, indicating that thiourea effectively inhibits the degradation of the polymer chains, which results in a desirable long-term stability of polymer.

#### 2.1.2. Long-Term Stability at Elevated Flue Gas Pressure

**Figure 2:**
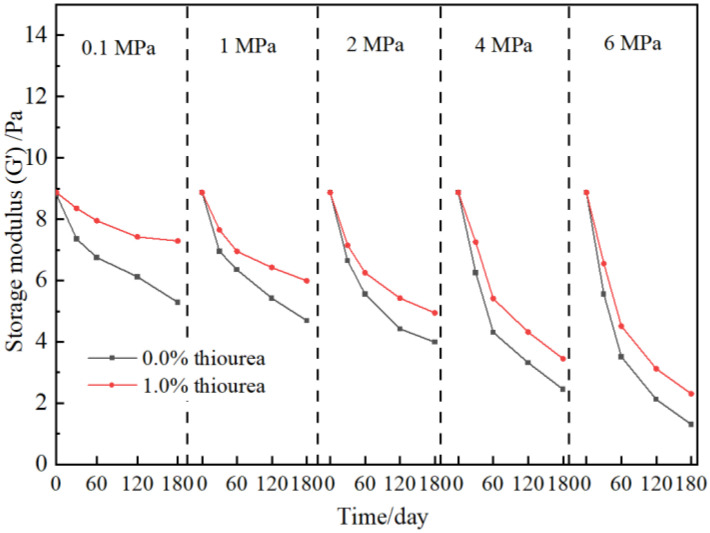
Effect of thiourea on polymer gel stability at elevated flue gas pressures.

According to the above result, the presence of thiourea may contribute to the long-term stability of cross-linked gels. The strength of polymer gel at elevated flue gas pressures is investigated. The gel formula, consisting of 0.5 wt% polymer and 0.0625 wt% chromium acetate, is optimized in our previous work and employed in this study [11,36]. 1.0 wt% thiourea is added to the gels. As shown in Figure 2, with the addition of thiourea, the strength and stability of the gels are all improved at elevated flue gas pressures. In detail, the storage modulus (G′) of the gel in the absence of thiourea decreases from 5.29 Pa to 1.31 Pa when the flue gas pressure rises from 0.1 MPa to 6 MPa after aging for 180 days while increasing from 7.92 Pa to 2.31 Pa in the presence of thiourea. This is because the thiourea inhibits the degradation of polymers in the gels, which in turn improves the long-term stability of bulk gels.

### 2.2. Nano-SiO_2_ Reinforced Polymer Gel

#### 2.2.1. Polymer Stability

**Figure 3:**
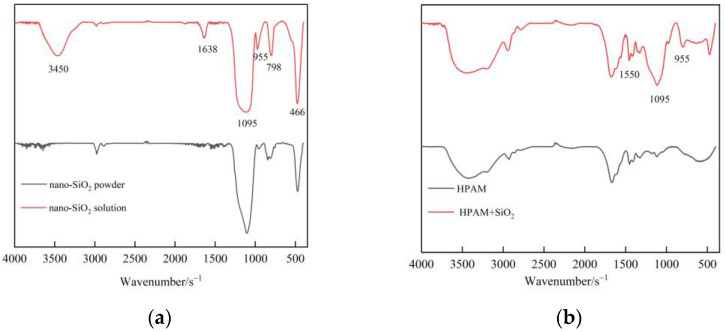
FTIR spectra of nano-SiO_2_ and polymer: (**a**) Nano-SiO_2_; (**b**) Nano-SiO_2_ and polymer.

Nano-SiO_2_ is an effective means to improve polymer stability and gel strength. The interaction between nanoparticles and polymers is studied by FTIR spectra analyses. Figure 3a contrasts the FTIR spectra of nano-SiO_2_ powder and nano-SiO_2_ solution. The strong and broad absorption band at 1095 cm^−1^ and the peaks at 798 cm^−1^ as well as 466 cm^−1^ in pure nano-SiO_2_ powder are attributed to the Si–O–Si antisymmetric stretching vibration and the Si–O bond symmetric stretching vibration. Afterwards, the nano-SiO_2_ is dispersed in an aqueous solution to form silica sol. The structural water –OH antisymmetric stretching vibration and the water H–O–H bending vibration appear at 3450 cm^−1^ and 1638 cm^−1^. The bending vibration peak of Si–OH appears at 958 cm^−1^. The absorption peaks of new groups indicate that the Si–O–Si on the surface of SiO_2_ decomposes into Si–OH in solution, which makes the nano-SiO_2_ carry a large number of –OH. After the addition of SiO_2_ nanoparticles to the polymer (Figure 3b), Si–NH_2_ bending vibration peaks appear between the nanoparticles and the HPAM at 1550 cm^−1^, respectively, which is consistent with the result from Giraldo et al. [22]. The –OH group in the 800 cm^−1^ region may react with amide (–NH_2_), aldehyde, and/or carboxylic acid groups (–COOH) to form hydrogen bridges. The interaction between functional groups was confirmed by FTIR analyses, where the –OH on the surface of the nano-SiO_2_ formed bridges and/or hydrogen bonds with the free groups O (amide group, carboxylic acid) and N (1550–1530 cm^−1^) of the polymer [37].

**Figure 4:**
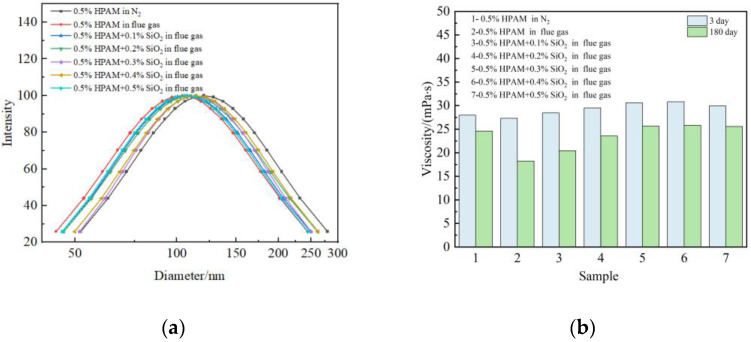
Effect of nano-SiO_2_ on polymer properties: (**a**) Hydrodynamic radium (R_h_); (**b**) Viscosity.

To further illustrate the effect of nano-SiO_2_ on the properties of polymer molecules, research was performed about R_h_ and viscosity changes of polymer solutions saturated with flue gas. Figure 4a shows the results of R_h_ changes in a 0.5 wt% polymer solution after aging for 3 days at 42 °C. The mass fraction of the nanoparticles ranged from 0 wt% to 0.5 wt%. It can be seen that the flue gas curves the polymer molecule chains and decreases the R_h_, which is founded in our previous work [11,12]. After the addition of nano-SiO_2_, the interaction between nano-SiO_2_ and polymer leads to an increase in R_h_ (Figure 4a), and improves the viscosity of the polymer solution (Figure 4b). It is worth noting that the viscosity increases distinctly with the addition of 0.0–0.3 wt% nano-SiO_2_. The polymer molecules are induced to form a uniform structure with the addition of 0.3 wt% nano-SiO_2_. Increasing the quantity of nano-SiO_2_ has a small influence on the steady structure of polymers, which results in a slight rising tendency when the concentration increases to 0.4 wt% or more. After aging for 180 days under the flue gas environment, the polymer solution still maintains excellent stability. The result demonstrates that the nano-SiO_2_ inhibits the entanglement of the polymer molecules and makes the polymer molecules more stretched, facilitating the dispersion of polymer molecules and the stability of polymer configuration.

#### 2.2.2. Gelation Performance

**Figure 5:**
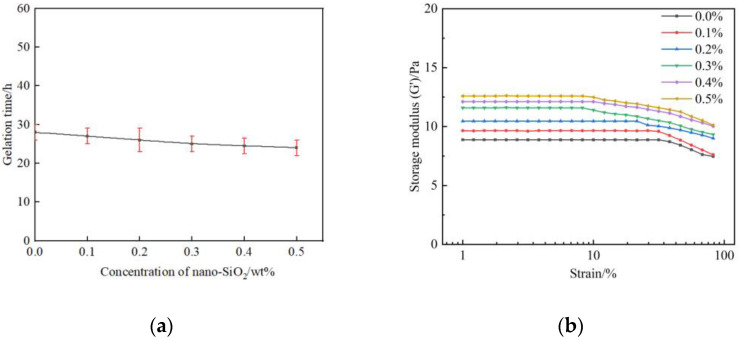
Effect of nano-SiO_2_ on gelation performance of polymer gel: (**a**) Gelation time; (**b**) Gel strength.

In order to specify the effect of nano-SiO_2_ on the gelation performance and gel strength, gelant samples consisting of different mass fractions of nano-SiO_2_ are prepared and then saturated with flue gas. The other component of gelant includes 0.5 wt% polymer, 0.0625 wt% chromium acetate, and 1.0 wt% thiourea. As shown in Figure 5a. The gelation time is shortened with the mass fraction of the nano-SiO_2_. Whereas, the trend is not obvious due to the fact that the gelation time is mainly dominated by the cross-linker behavior [36]. The strength of gels after aging for three days is further investigated. It can be obtained from Figure 5b that the G′ of gel in the absence of nano-SiO_2_ was about 8.87 Pa and rises to 9.65–12.5 Pa when the mass fraction of nano-SiO_2_ increases to 0.1–0.5 wt%. The gel strength has been significantly improved with the introduction of the nanoparticles.

#### 2.2.3. Long-Term Stability at Elevated Flue Gas Pressure

**Figure 6:**
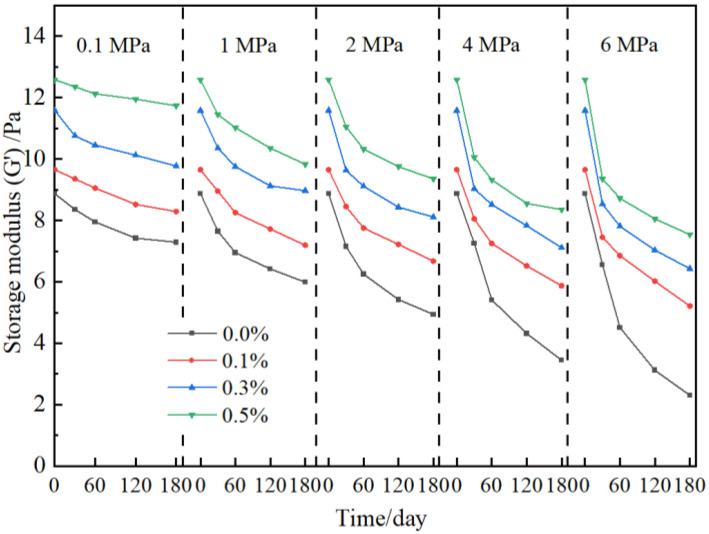
Effect of nano-SiO_2_ on gel stability at elevated flue gas pressures.

To study the influence of nanoparticles on the long-term stability of gels, the strength of gel under high-pressure flue gas is further investigated, as shown in Figure 6. The strength of gel without the addition of nano-SiO_2_ decreases to different degrees under different flue gas pressures and performs seriously with the increase in pressure due to the comprehensive effect of CO_2_ and O_2_ composed in flue gas [11,12]. In contrast, the strength of the gel in the presence of nano-SiO_2_ is improved under different flue gas pressures. With the increase in nano-SiO_2_ concentration, the gel strength increases obviously. Taking 2 MPa of flue gas as an example, the storage modulus of gels without nano-SiO_2_ in a flue gas environment for 180 days is 4.94 Pa, while with the addition of 0.1%, 0.3%, and 0.5% nano-SiO_2_, the increase percentage of gel strength is calculated to be 25.9%, 39.1%, and 47.2%, respectively. In our previous work, it was also found that the nano-SiO_2_ reinforced gel showed satisfying long-term stability and maintained an intact network structure [38].

#### 2.2.4. Structure of Gel

**Figure 7:**
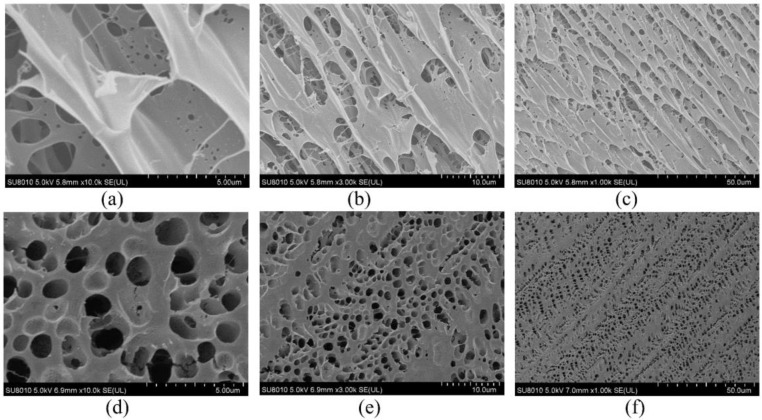
Cryo-SEM image of polymer gel: without nano-SiO_2_: (**a**) ×10.0 k; (**b**) ×3.0 k; (**c**) ×1.0 k; with 0.3 wt% nano-SiO_2_: (**d**) ×10.0 k; (**e**) ×3.0 k; (**f**) ×1.0 k.

**Figure 8:**
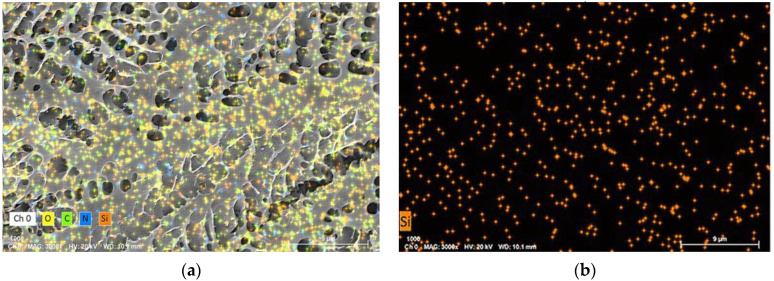
SEM-EDS image of the polymer gel: (**a**) Distribution of O, C, N and Si element in the gel without the addition of SiO_2_; (**b**) Distribution of Si element in the gel with the addition of 0.3 wt% SiO_2_.

To further study the stability mechanism of reinforced gel, a Cryo-SEM is conducted to investigate the microstructures of gels. The gel sample group is prepared with 0.5 wt% polymer, 0.0625% chromium acetate, 1.0 wt% thiourea, and 0.3 wt% nano-SiO_2_. Notably, the optimized concentration of nano-SiO_2_ is 0.3 wt% as a result of acceptable gel strength and economic consideration. The blank group is in absence of nano-SiO_2_. The gels are aged for 180 days in 2 MPa flue gas. As compared in Figure 7, it is found that the blank group has a heterogeneous lamellae structure with many holes due to the fact that the flue gas penetrates the gel. The cracks observed in the gel lead to a decrease in G′, as shown in Figure 7a–c. Whereas, for the gel sample group, the gel exhibits a honeycomb structure, presenting a firm and uniform network structure (Figure 7d–f). Noteworthily, the pore size of the gel with the addition of nano-SiO_2_ is 1–2 um, whereas it varies from 2 um to 10 um in the blank sample. Based on the result from Section 2.2.3, it is hypothesized that the decrease in pore size leads to a reduction in surface area and an increase in gel density, which positively improves gel strength. Furthermore, the Croy-SEM-EDS images indicate that the gel network structure is uniformly interspersed with the nano-SiO_2_ particles (orange dots in Figure 8). In conclusion, hydrogen bonding is formed between nano-SiO_2_ and polymer molecules, which makes them more stretched in the flue gas environment and promotes the homogeneity of the polymer. Furthermore, the adsorption of nano-SiO_2_ onto polymer chains encrypts the network structure of bulk gel. The enhancement of microstructure leads to an increase in the viscosity of the gelant and the strength of the cross-linked gel, which greatly improves the long-term stability of the gel.

### 2.3. Creep Behaviour of Polymer Gel

**Figure 9:**
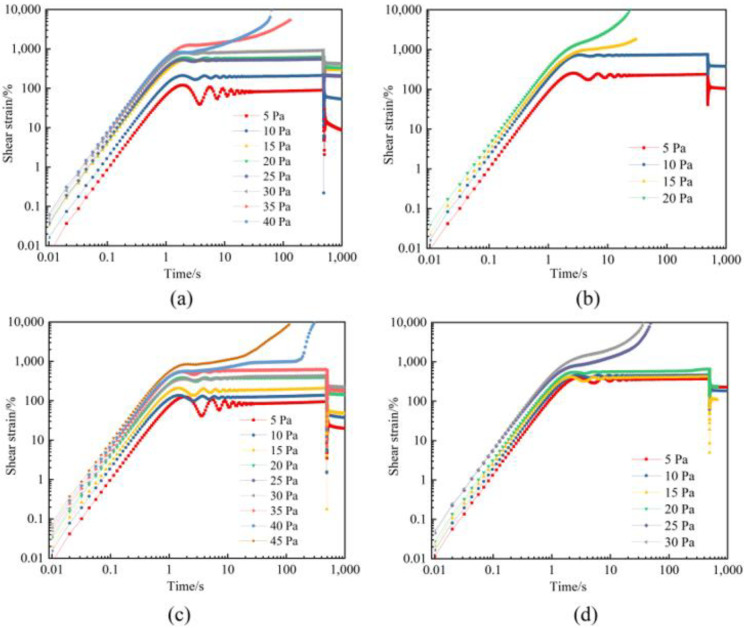
Creep strain of polymer gels at different shear stresses: (**a**) Gel without the additives after 3 days; (**b**) Gel without the additives after 180 days; (**c**) Gel with the addition of additives after 3 days; (**d**) Gel with the addition of additives after 180 days.

Creep and creep recovery tests are carried out to evaluate the compression resistance of gels in a flue gas environment. Figure 9 depicts the creep curves of polymer gels without and with additives, including nano-SiO_2_ and thiourea. Under a constant stress far below the elastic limit, the gel increases linearly for a few seconds, then yields, the strain decreases and oscillates, and thereafter the strain flattens10 or increases sharply. It can be seen that with the increased aging time under high-pressure flue gas, the failure stress of gels decreases. The initial failure stress of gels is 30 Pa, and then decreases to 10 Pa after aging for 180 days (Figure 9a,b). In contrast, the initial failure stress of gel with the addition of additives is greater than 35 Pa and decreases to 20 Pa after aging for 180 days (Figure 9c,d). The polymer gel still maintains its robust structure under extensive deformation. It is indicated that thiourea and nano-SiO_2_ improve the compressive properties of gels.

**Figure 10:**
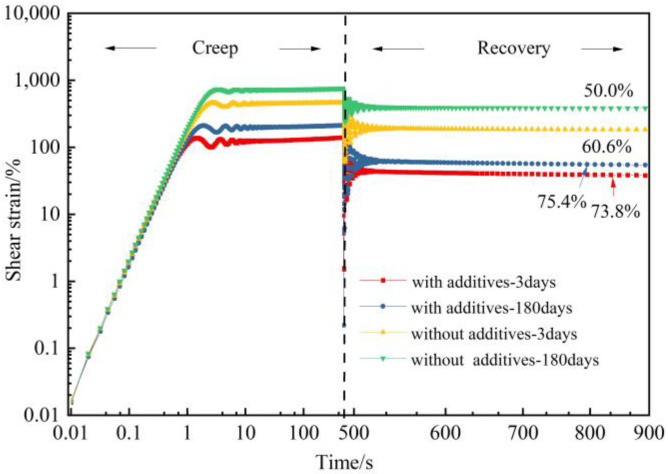
Creep and creep recovery test of polymer gel with and without addicatives.

Creep-recovery experiments can clearly distinguish the elastic and viscous responses of gels. The creep recovery of gels at 10 Pa stress was investigated. It can be seen from Figure 10 that after unloading by external forces, part of the deformation is recovered due to the elasticity of the gels, and the other part of the deformation is lost due to flow. After aging for 180 days under 2 MPa flue gas pressure, the creep recovery rate of gels decreases from 60.6% to 50.0%. With the addition of additives to the gel system, the creep compliance was reduced, and the creep recovery rate of the gel decreased from 75.4% to 73.8%. The results indicate that the introduction of additives increases the elasticity of polymer gels, which may be caused by the interaction between nano-SiO_2_ and polymers and the inhibited degradation resulting from thiourea.

### 2.4. Flow Experiment

#### 2.4.1. Injection Performance

**Figure 11:**
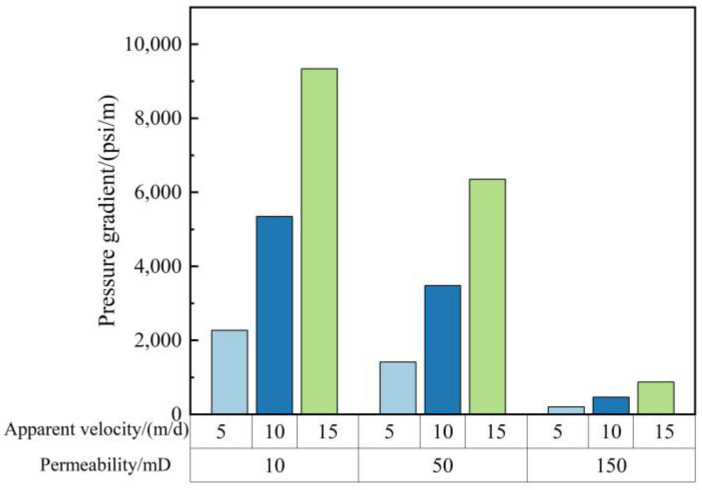
Injection performance of gelant in different permeability and injection velocity.

Well Hong 48 in Xingjiang oilfield is a low-permeability (10–500 mD) flue gas flooding reservoir, which requires good injection performance of gelant. The injection performance of gelant in a low-permeability core is investigated. Sandstone cores, with a permeability of 10 mD, 50 mD, and 150 mD, are prepared. The gelant, consisting of 0.5 wt% HPAM, 0.625 wt% chromium acetate, 0.3 wt% nano-SiO_2_, and 1% thiourea, was employed for the three flooding experiments. Figure 11 depicts the relationship between the injection pressure gradient and the permeability as well as the injection velocity of gelant. Under a certain injection speed, the injection pressure gradient decreases with the increase in permeability. The injection pressure gradient was acceptable in 50 mD and 150 mD cores. Although the value was relatively high in the 10 mD core, no blocking phenomenon was found during the gelant flooding experiment. The result shows that the gelant composed of a 2 million molecular weight polymer has good injection performance.

#### 2.4.2. Plugging Performance

**Figure 12:**
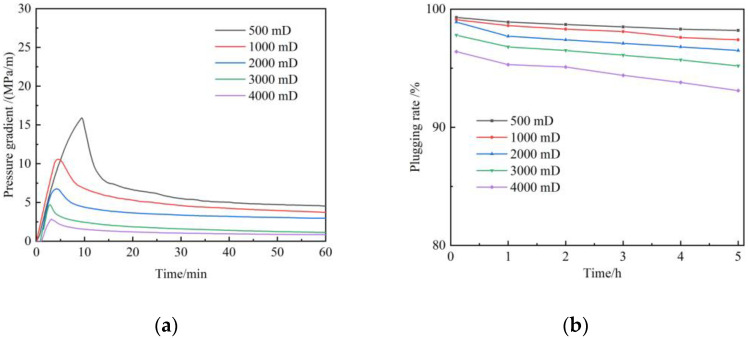
Plugging performance of polymer gel during flue gas flooding: (**a**) Breakthrough pressure gradient; (**b**) Plugging rate.

The average permeability of the target region is 58 mD, with a high permeability layer and serious heterogeneity. The parameters and heterogeneity characteristics of interwell channels are analyzed using tracer monitoring technology. The results show that the permeability ranges from 78.9 mD to 6362.5 mD in the monitoring area. Based on the principle of division of dominant channels, inter-well channels are divided into three sections, namely, first-level dominant channels, second-level dominant channels, and third-level dominant channels (Table 1). The primary dominant channel accounted for 15.4%, with an average permeability of 2432 mD. Secondary dominant channels accounted for 35.9%, with an average permeability of 796 mD. The primary and secondary channels are scheduled to be plugged. Hence, the plugging performance of gels in a 500–4000 mD reservoir was investigated with the sandpack flow experiment. Figure 12a depicts the relationship between the breakthrough pressure of gel with the permeability of sandpacks during the flue gas flooding. The breakthrough pressure gradient of gel decreases with the increase in permeability. The breakthrough pressure gradient can reach from 2.8 MPa/m to 15 MPa/m when the permeability of sandpacks increases from 500 mD to 4000 mD. Figure 12b shows the plugging rate as a function of time after the gel breakthrough. It can be seen that the gel shows good plugging performance and scouring resistance in high-permeability regions. The plugging rate can still be maintained above 93% after 5 h. Therefore, the gel is recommended to plug the primary channel and secondary channel in the target region.

#### 2.4.3. Mechanism of Gel Treatment

**Figure 13:**
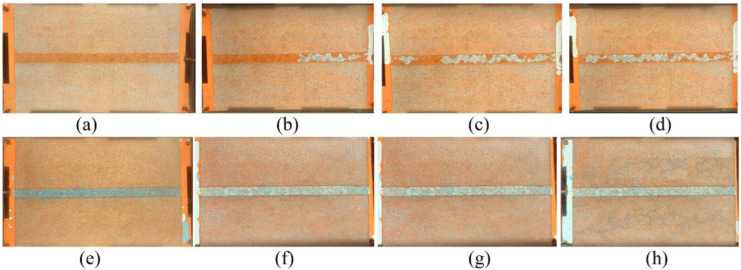
Flue gas flooding in visual glass plate model: (**a**) saturated with oil; (**b**) initial stage of gas flooding;(**c**) intermediate stage of gas flooding; (**d**) final stage of gas flooding; (**e**) polymer gel formed in the high permeability area; (**f**) initial stage of subsequent gas flooding;(**g**) intermediate stage of subsequent gas flooding; (**h**) final stage of subsequent gas flooding.

In order to observe the formation mechanism of gas channeling in flue gas flooding and evaluate the plugging performance of polymer gel, a visual glass plate model is employed with a 7:1 permeability ratio, as shown in Figure 13. The visual glass plate model is prepared by saturating it with oil (Figure 13a). Furthermore, flue gas is continuously injected into the model. During flue gas flooding, gas channeling occurs in the high permeability area, and the saturated oil is displaced easily (Figure 13b–d). Flue gas is incapable of spreading to low-permeability areas, leading to no increase in oil recovery. The gelant is injected and filled in the high permeability area. Then it is aged for 3 days in a 42 °C oven to form a bulk gel (Figure 13e). The flue gas flooding is performed afterwards. It is obviously found that the gas spreads to the low-permeability area. The oil is displaced out of the low-permeability area, and the recovery efficiency is significantly improved (Figure 13f–h). The phenomenon indicated that the polymer gel has a perfect plugging capability in flue gas flooding reservoirs.

#### 2.4.4. Design of Field Application

Firstly, the volume of gelant is designed according to the plugging radius. The calculation formula is as follows:

*V* = π × *R*^2^ × *H × φ* × *β* (4)

where *V* is the volume of gelant, *R* is the plug radius, *H* is the thickness of the formation, *φ* is the porosity, %; *β* is the comprehensive coefficient. Meanwhile, the plug radius is generally designed to be 5–10 m for the near-well profile control. The plug radius is designed to be about 50 m, or even 1/4–1/3 of the well distance, for in-depth well profile control. The amount of gelant is designed to be 40% of the volume of the first-level dominant channel, and 30% of the volume of the second-level dominant channels. The details of the volume of gelant are shown in Table 2. After the injection of the gelant, the follow-up displacement slugs, i.e., 0.2 wt% or 0.3 wt% polymer solution, should be exactly controlled to make sure that all the gelant is injected into the target area. Afterward, the wells are shut-in for 3 days. Then the wells are opened, and the flue gas flooding is conducted. The design of gel treatment can provide guidance for flue gas mobility control in Xinjiang oilfield.

## 3. Conclusions

In this study, a reinforced gel system for mobility control in flue gas flooding reservoirs was proposed. Thiourea and nano-SiO_2_ were employed as reinforcing additives to improve the long-term stability of polymer gels. Based on the experimental results, the following conclusions can be drawn:

1. Thiourea, which acts as an oxygen scavenger, inhibits the degradation of polymers in the gel, which in turn improves the long-term stability of bulk gel at elevated flue gas pressures.
2. The interaction between nano-SiO_2_ and HPAM molecules by hydrogen bonding inhibits the entanglement of the polymer chains, which facilitates the dispersion and the long-term stability of polymers in flue gas environments.
3. The gel with the addition of nanoparticles shows a firmer and more uniform microstructure compared with the traditional gel system. In addition, the additives improve the compressive properties of the gel, which still maintains its robust structure under the extensive deformation. As a result, the strength and long-term stability of gels are effectively improved under high pressure of flue gas in 180 days.
4. Flow experiments indicate that the reinforced gel system is an effective plugging agent with satisfying scouring resistance, which is suitable for flue gas flooding reservoirs.

## 4. Materials and Methods

### 4.1. Materials

Partially hydrolyzed polyacrylamide (HPAM) was supplied by SNF Flocculant, Co. Ltd., Taixing, China. The molecular weight of the HPAM is 2 million, and the hydrolysis degree is 4%. Chromium acetate (50 wt%), thiourea (99% purity), and nano-SiO_2_ were obtained from Sigma-Aldrich, Shanghai, China. In particular, the particle size of nano-SiO_2_ is 20 nm, and the solid content is 30%. High purity N_2_ and flue gas (80% N_2_, 17% CO_2_, and 3% O_2_) were supplied by Shanghai Shenkai Gas Co. Ltd., Shanghai, China. The polymer gels are prepared with formation water, and the composition was presented in the previously published paper [12].

### 4.2. Experiment Methods

#### 4.2.1. Gel Preparation and Evaluation

The gelant was formulated by even mixing HPAM stock solution, cross-linker solution, and additives, including chromium, thiourea, and nano-SiO_2_, in different proportions. Then the gelant was placed at 42 °C (reservoir temperature) for cross-linking. The specific preparation method was elaborated in the previously published article [12]. The properties of gel, including gelation time and the strength of the polymer gels, were determined by the G′. The G′ was measured by Anton Paar rheometer with a strain amplitude ranging from 1% to 500% at a frequency of 1 Hz.

The creep-recovery test was also conducted by the Anton Paar rheometer. In the creep stage, a constant shear stress was set. In the creep recovery stage, shear stress was set to 0. The relationship between shear strain and time was recorded in the whole test process.

#### 4.2.2. Characterization

The R_h_ of polymer molecules were measured on a Nano Brook 90 plus DLS (Brookhaven, New York, NY, USA) instrument and adopted to determine the configuration variation of the polymer.

FTIR spectra analyses were performed on a Nexus FTIR spectrometer (Nicolet, Madison, WI, USA) over a range of 4000–400 cm^−1^ and adopted to characterize the composition and transformation of nano-SiO_2_.

The SEM equipped with a SU8010 Cryo-SEM (Hitachi, Tokyo, Japan) and EDS was adopted to investigate the microstructure of the polymer gel in a frozen state.

#### 4.2.3. Flooding Test

**Figure 14:**
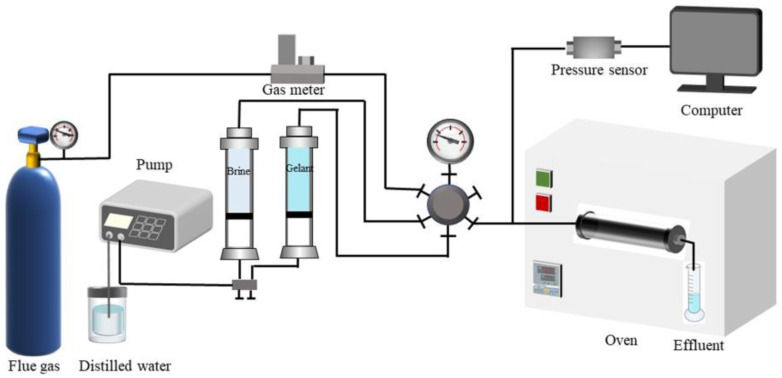
Schematic of equipment for investigating the plugging performance of gel.

The injection performance of gelant was characterized by the injection pressure gradient and was determined by the flow experimental device, as shown in Figure 14. Firstly, sandstone cores with a 2.5 cm diameter and 10 cm length were prepared and saturated with formation water, and then flue gas was injected into the cores until the injection pressure held steady. Finally, gelant was injected at different injection rates. Meanwhile, the injection pressure was recorded. The injection pressure gradient was calculated afterwards.

The plugging ability of gels was characterized by the breakthrough pressure gradient and plugging rate. Firstly, sandpack cores were made with different meshes of quartz sand and saturated with formation water. Flue gas was injected afterwards. Next, gelant was injected into the sandpack and kept in a 42 °C oven to form bulk gel. Finally, the flue gas was injected at a certain rate. The injection pressure should be recorded throughout the experiment. The breakthrough pressure gradient and plugging rate were calculated.

#### 4.2.4. Visual Glass Model Experiment

The visual glass model displacement experiment was carried out using a self-made glass model that was made according to reservoir conditions. The glass model was 15 cm width and 20 cm length. A high-permeability area was designed by filling with 30 mesh number sand in the middle of the glass model, and the remaining area was filled with 60–70 mesh number sand. Then it was sealed and compacted with rubber rings around it. The process of flue gas flooding and gel treatment was recorded by a Canon camera.

## Figures and Tables

**Table 1:** Parameters of channels type.

Channel Type	Thickness/m	Range of PermeAbility/mD	Mean PermeAbility/mD	Volume /m^3^	Proportion /%
First-level	1.29	2021.6–6362.5	2432	1979.75	15.4
Second-level	1.16	416.4–1576.1	796.3	1741.89	35.9
Third-level	0.53	78.9–317.7	190.2	826.29	48.7

**Table 2:** Designed volume of gelant.

Well	First-Level Dominant Channel		Second-Level Dominant Channel	
	Volume of Channel /m^3^	Volume of Gelant /m^3^	Volume of Channel /m^3^	Volume of Gelant /m^3^
1	5729	2292	4704	1411
2	920	368	1697	509
3	3608	1443	4933	1480
4	6900	2760	7528	2258

## Data Availability

Not applicable.
